# The Impact of Faculty Gender on Resident Evaluations of Faculty Performance in Emergency Medicine

**DOI:** 10.7759/cureus.56814

**Published:** 2024-03-24

**Authors:** Allison M Beaulieu, Katherine M Hunold, Jennifer Mitzman, Simiao Li-Sauerwine

**Affiliations:** 1 Emergency Medicine, The University of Utah, Salt Lake City, USA; 2 Emergency Medicine, The Ohio State University Wexner Medical Center, Columbus, USA; 3 Pediatrics/Emergency Medicine, Nationwide Children’s Hospital, Columbus, USA

**Keywords:** emergency medicine, medical education research, medical education evaluation, gender, medical education

## Abstract

Introduction: Gender bias impacts the promotion and tenure of female emergency medicine (EM) physicians and limits their ability to advance in academic rank. Many factors influence the promotion and tenure process including research, evaluations, opportunities for leadership, sponsorship, and mentorship. The goal of this study is to determine if resident evaluations of EM faculty differ by faculty gender.

Methods: A quantitative analysis was used to examine 14,613 teaching evaluations of faculty by residents at a single academic center (The Ohio State University Wexner Medical Center, Columbus) in the years 2017-2019. Anonymized ratings of male and female faculty on a five-point Likert scale were compared using Fischer’s exact test and adjusting for multiple comparisons.

Results: Male faculty were more likely to hold the rank of Associate Professor or Professor. When taking both faculty gender and rank into account, male Clinical Instructors and Assistant Professors had significantly higher evaluation scores by residents in the domain of resident autonomy than their female counterparts. Regardless of gender or faculty rank, the majority of faculty received scores greater than four.

Conclusion: A significant gender difference was found in resident evaluation scores of faculty in the domain of resident autonomy at the level of Clinical Instructor and Assistant Professor. Resident autonomy refers to the degree of supervision by faculty which evolves over time and is primarily based on level of training. This is important as it demonstrates a gender difference in scores that could be used to determine faculty compensation and promotion. Evaluation tools used for promotion and tenure of academic faculty should be evaluated for implicit bias and appropriate statistical analysis.

## Introduction

Promotion and tenure in academic medicine are dependent on a variety of accomplishments including the number of publications, grants, awards, and leadership positions [1-3]. Female academic physicians tend to rank below male peers in each of these standardized categories as compared to their male colleagues [3]. Various factors contributing to implicit gender bias have led to female physicians producing fewer publications, obtaining less research funding, achieving fewer leadership positions, and receiving weaker letters of evaluations [4-6]. Therefore, female physicians advance to the next academic rank at lower rates and thus receive a lower salary as compared to their male counterparts [7-10].

When investigating the specialty of emergency medicine (EM), the differences in academic rank persist and female academic EM physicians are less likely to hold higher ranking academic positions such as Associate or Full Professor [4,5,10]. While it has been established that lack of research funding, mentorship, sponsorship, and letters of recommendation contribute to this trend, it is important to identify other contributing factors that lead to this discrepancy within the promotion and tenure process [4-6,11]. One such additional factor that is considered in the promotion and tenure process, especially for those in Clinician-Educator Tracks, is the education portfolio [12]. An education portfolio is commonly composed of publications, grants, lectures, committee service, and documentation of teaching excellence [12]. The documentation of teaching excellence, which directly impacts the promotion and tenure process, consists of evaluations by trainees such as students, residents, and fellows [12]. Resident and trainee evaluations may play an important role in demonstrating excellence in teaching and mentorship.

The current literature supports that female faculty generally receive lower evaluation scores from trainees than their male colleagues [13-16]. These differences are further exacerbated in male-dominated fields such as surgery but also exist in female-dominated fields such as pediatrics [15,16]. Female pediatricians received lower scores in both teaching and role modeling than their male counterparts [16]. Another study demonstrated multiple gender-based differences in the assessment of general internal medicine faculty physicians by trainees [11]. Male faculty were rated more highly in multiple categories including ability to teach, medical knowledge, professionalism, practice-based learning and improvement, and systems-based practice [17]. The only category female internal medicine physicians scored higher than their male colleagues was outpatient patient care [17]. This is relevant as EM physicians play a vital role in patient education, counseling, and safe discharge similar to physicians who practice in an outpatient setting.

The existence and extent to which learner evaluations of faculty in EM differ based on faculty gender are not well characterized. The purpose of this study is to determine the impact of faculty gender on the evaluation of academic EM physicians.

This article was previously presented as a meeting abstract at the 2021 SAEM Annual Scientific Meeting on May 12, 2021 and 2021 IAMSE Annual Scientific Meeting on June 15, 2021.

## Materials and methods

Study design

We performed a retrospective study comparing numeric scores of faculty performance in the clinical setting from evaluation forms completed by residents. This study was deemed exempt by the institutional review board at The Ohio State University.

Study setting and population

All resident evaluations of EM faculty at a single three-year ACGME-accredited residency program at a large academic medical center (The Ohio State University Wexner Medical Center, Columbus) in the Midwest from January 1, 2018, to December 31, 2019, were included in analyses. For each 28-day educational block in the emergency department (ED), resident physicians are required to complete at least 10 evaluations of faculty members with whom they worked clinically in that time period. The resident physicians are assigned evaluations for each shift and can choose which faculty to evaluate. Therefore, they can evaluate a faculty member more than once during each block only if they worked more than one clinical shift with that faculty member during the 28-day block. All residents in the program (n=72) completed evaluations in this time period. Each evaluation consisted of a Likert scale in five domains as well as an overall score (Table 1). The evaluation tool created using the Accreditation Council for Graduate Medical Education (ACGME) creates Common Program Requirements (CPR) for EM [18]. 

**Table 1:** Areas of teaching ability evaluated by emergency medicine residents for emergency medicine faculty.

Domain	Description
Autonomy	This attending provided appropriate autonomy and supervision of trainees in direct patient care and bedside procedures.
Discussion	This attending promoted case-specific discussion to trainees on illness presentation or patient management.
Feedback	This attending provides feedback on clinical skills, clinical reasoning, or patient management to trainees.
Engagement	Degree of engagement (availability, approachability, dedication to teaching and patient care) exhibited by this attending to trainees.
Professionalism	This attending models professional behavior through communication with all members of the patient care team for trainees.
Overall	Overall teaching effectiveness of this attending to trainees.

Faculty members in this study were identified by gender and academic rank to account for potential differences by professorial level. During the study period, there were 66 faculty members: 27 females and 39 males. Eight faculty members were promoted during the study period and their evaluations were analyzed with the group to which they belonged at the time of the evaluation. To preserve the anonymity of a single male Clinical Instructor and single female Full Professor, Clinical Instructors and Assistant Professors were combined, and Associate and Full Professors were combined for all analyses. Thus, there were a total of 74 faculty levels for evaluation.

Sample size

All available evaluations were utilized which included 14,613 evaluations. Thus, no sample size calculation was performed. However, for our comparisons of proportions and this sample size, we anticipated having 80% power to detect a difference as small as 2% between male and female faculty. Were we to have 50% fewer evaluations than anticipated (6500), we would have 80% power to detect a difference as small as 3%. Thus, we determined that our sample size was sufficient for the proposed analysis.

Analysis

Academic rank by gender was compared using Fischer’s exact test with a p-value of 0.05. Fischer’s exact test was also applied to examine for gender differences in resident evaluations in each of the six questions among Clinical Instructors and Assistant Professors (Clinical Instructor/Assistant) and Associate and Full Professors (Associate/Full). As this was the primary analysis, this is the only analysis adjusted for multiple comparisons with the Bonferroni correction and thus the p-value for significance was 0.0042.

A test of means or medians was not appropriate for this data due to extreme skew. A sensitivity analysis was done to examine for clustering among attending physicians. All data analyses were conducted in STATA 16 (StataCorp, College Station, TX).

## Results

During the study period, Clinical Instructors included one male and five females, Assistant Professors: 24 males and 19 females, Associate Professors: 10 males and five females, and Full Professors: nine males and one female. Male faculty were more likely to hold the rank of Associate/Professor than female faculty (Table 2, p < 0.05).

**Table 2:** Academic rank of emergency medicine faculty by gender at a single, academic center in the Midwest. *Indicates statistical significance by p-value <0.05.

	Clinical Instructor and Assistant Professor	Associate Professor and Professor*	Total
Female	24	6	30
Male	25	19	44
Total	49	25	74

A total of 14,613 evaluations were included in the study period which were completed by 72 residents. Among evaluations of Clinical Instructors and Assistant Professors (n=10,219), 4,397 were of female faculty and 5,822 were of male faculty. Among Associate and Full Professors (n=4,394), 1,003 were of female faculty and 3,391 were of male faculty.

Scores were highly clustered such that the majority of faculty received positive scores (≥4) across all questions regardless of faculty rank and gender. Accounting for multiple comparisons, we identified a significant difference in autonomy and overall teaching scores between male and female faculty (Table 3). When accounting for academic rank, autonomy was the only significant difference between evaluations of Clinical Instructors and Assistant Professors by gender (p<0.001, Table 4). In sensitivity analysis, there was clustering by faculty members with a small subset of low scores obtained by a small cohort of faculty, rather than being evenly distributed across the group. The sensitivity analysis demonstrated clustering by faculty members but did not change the overall results.

**Table 3:** Resident ratings of faculty members in response to six questions on a 5-point Likert Scale (1=strongly disagree / poor, 3=neutral / good, 5=strongly agree / excellent) by faculty gender. Data are presented as n and percentages with p-values for Fischer’s Exact Test with alpha=0.008 due to multiple comparisons.

	Female (n=5,400)	Male (n=9,213)	p-value
Overall			
5	3,019 (56.0)	5,459 (59.3)	<0.0001
4	1,942 (35.9)	3,152 (34.2)	-
3	350 (6.5)	482 (5.2)	-
2	77 (1.4)	102 (1.1)	-
1	12 (0.2)	18 (0.2)	-
Autonomy			
5	3,039 (56.3)	5,700 (61.9)	<0.0001
4	2,160 (40.0)	3,341 (36.3)	-
3	147 (2.7)	131 (1.4)	-
2	41 (0.8)	31 (0.3)	-
1	13 (0.2)	10 (0.1)	-
Discussion			
5	3,154 (58.4)	5,611 (60.9)	0.016
4	2,070 (38.3)	3,276 (35.6)	-
3	143 (2.7)	256 (2.7)	-
2	28 (0.5)	61 (0.7)	-
1	5 (0.1)	9 (0.1)	-
Feedback			
5	3,049 (56.5)	5,423 (59.0)	0.038
4	2,090 (38.7)	3,329 (36.1)	-
3	216 (4.0)	369 (4.0)	-
2	38 (0.7)	74 (0.8)	-
1	6 (0.1)	10 (0.1)	-
Engagement			
5	3,172 (58.7)	5,628 (61.1)	0.016
4	1,792 (33.2)	2,957 (32.1)	-
3	338 (6.3)	493 (5.4)	-
2	80 (1.5)	113 (1.2)	-
1	18 (0.3)	22 (0.2)	-
Professionalism			
5	3,285 (60.8)	5,713 (62.0)	0.065
4	1,783 (33.0)	3,016 (32.8)	-
3	273 (5.1)	415 (4.5)	-
2	52 (1.0)	56 (0.6)	-
1	7 (0.1)	13 (0.1)	-

**Table 4:** Resident ratings of faculty members in response to six questions on a 5-point Likert Scale (1=strongly disagree / poor, 3=neutral / good, 5=strongly agree / excellent) by faculty level and gender. Data are presented as n and percentages with p-values for Fischer’s Exact Test with alpha=0.0042 due to multiple comparisons. *Indicates statistical significance by Fischer's Exact Test with Bonferroni Correction (p<0.0042).

	Fellow/Assistant					Associate/Professor				
	Female (n=4,397)		Male (n=5,822)		p-value	Female (n=1,003)		Male (n=3,391)		p-value
Overall										
5	2,450	(55.7)	3,409	(58.6)	0.006	569	(56.7)	2,050	(60.5)	0.144
4	1,576	(35.8)	2,022	(34.7)	-	366	(36.5)	1,130	(33.3)	-
3	295	(6.7)	306	(5.3)	-	55	(5.5)	176	(5.2)	-
2	64	(1.5)	72	(1.2)	-	13	(1.3)	30	(0.9)	-
1	12	(0.3)	13	(0.2)	-	0	(0.0)	5	(0.2)	-
Autonomy										
5	2,450	(55.6)	3,595	(61.8)	<0.001*	589	(58.7)	2,105	(62.1)	0.220
4	1,770	(40.3)	2,129	(36.6)	-	390	(38.9)	1,212	(35.7)	-
3	129	(2.9)	73	(1.3)	-	18	(1.8)	58	(1.7)	-
2	35	(0.8)	19	(0.3)	-	6	(0.6)	12	(0.4)	-
1	13	(0.3)	6	(0.1)	-	0	(0.0)	4	(0.1)	-
Discussion										
5	2,586	(58.8)	3,487	(59.9)	0.130	568	(56.6)	2,124	(62.6)	0.008
4	1,670	(38.0)	2,113	(36.3)	-	400	(39.9)	1,163	(34.3)	-
3	115	(2.6)	170	(2.9)	-	28	(2.8)	86	(2.5)	-
2	21	(0.5)	46	(0.8)	-	7	(0.7)	15	(0.4)	-
1	5	(0.1)	6	(0.1)	-	0	(0.0)	3	(0.1)	-
Feedback										
5	2,484	(56.5)	3,359	(57.7)	0.045	565	(56.3)	2,064	(60.9)	0.020
4	1,711	(38.9)	2,144	(36.8)	-	379	(37.8)	1,185	(35.0)	-
3	170	(3.9)	250	(4.4)	-	46	(4.6)	119	(3.5)	-
2	26	(0.6)	55	(0.9)	-	12	(1.2)	19	(0.6)	-
1	5	(0.1)	6	(0.1)	-	1	(0.1)	4	(0.1)	-
Engagement										
5	2,590	(58.9)	3,558	(61.1)	0.015	582	(58.0)	2,070	(61.0)	0.309
4	1,442	(32.8)	1,880	(32.3)	-	350	(34.9)	1,077	(31.8)	-
3	287	(6.5)	304	(5.2)	-	51	(5.1)	189	(5.6)	-
2	62	(1.4)	65	(1.1)	-	18	(1.8)	48	(1.4)	-
1	16	(0.4)	15	(0.3)	-	2	(0.2)	7	(0.2)	-
Professionalism										
5	2,689	(61.2)	3,556	(61.1)	0.144	596	(59.4)	2,157	(63.6)	0.090
4	1,437	(32.7)	1,955	(33.6)	-	346	(34.5)	1,061	(31.3)	-
3	224	(5.1)	270	(4.6)	-	49	(4.9)	145	(4.3)	-
2	40	(0.9)	31	(0.5)	-	12	(1.2)	25	(0.7)	-
1	7	(0.2)	10	(0.2)	-	0	(0)	3	(0.1)	-

## Discussion

Female faculty at the clinical instructor and assistant professor level were rated as allowing less autonomy and providing more supervision in direct patient care and bedside procedures than their male faculty counterparts of the same rank. According to the ACGME CPR, autonomy refers to the appropriate supervision of residents by attendings [18]. Supervision is essential for patient safety and high-quality teaching [18]. The degree of resident autonomy, balanced with supervision, progresses with the level of training and attainment of various skills within the field of EM [18]. It is also affected by various clinical factors such as acuity, complexity, urgency, and risk of serious or adverse events [18]. While prior studies of autonomy with respect to teacher gender are not available in EM, in prior studies in surgical specialties, female residents were given less autonomy than male residents by male faculty, while no statistical difference in resident autonomy was found when working with female faculty [19,20]. In a qualitative study investigating factors that impact resident autonomy in EM, several themes were found that require the attending to provide more supervision and direct patient care [21]. These include caring for families who request additional care and services, environmental factors such as patient volume and system protocols, resident level and experience, and faculty confidence [21]. 

An additional finding was the non-normal distribution of data. The majority of faculty members received scores greater than four across all categories. The Likert Scale had a rating system of one - strongly disagree to five - strongly agree. Thus, if a resident agreed with the statement, they were likely to choose a score of 4 or higher. Given the distribution of evaluations for all faculty members, there is no meaningful distinction between excellent educators and more average educators within the department. This could be influenced by many factors including socio-behavioral elements of the residents completing the scoring, an evaluation form not attuned to delineate differences between “good” and “excellent” teaching, or may indicate a high level of teaching skill by faculty in the department. This ceiling and floor effect has been previously delineated in the literature; deliberate instruction in use of evaluation forms may limit bias [22]. At our institution, teaching scores are reported as means. It is important that department chairs and promotion and tenure committees discern whether mean scores of faculty rankings are a valid marker of performance in a sample with a non-normal distribution.

Limitations of this study include that data was collected from a single academic department of EM; resident gender, level of training, and competency level were not collected; and that it was subject to skewed scoring. Residents were assigned an evaluation for each shift and must complete ten per block of EM which is an administrative requirement tracked by the residency. It is unknown how residents chose which 10 attendings they wish to evaluate which may have affected our results. The gender of residents was not collected, as the evaluations were anonymous with respect to the learner, and thus an analysis of faculty rating based on learner gender was not possible. The overall gender of the residents in the program ranged from 29% female in 2017, to 58% female in 2018-19. Since completion is a requirement, this should represent the approximate balance of the respondents.

To improve distribution of data and obtain more meaningful evaluations, a continuous scale rather than a Likert scale may be used in future faculty evaluations. We also recommend active steps to mitigate implicit gender bias in the evaluations of academic female faculty by residents including evaluator training, structured evaluation forms, and an evaluation guide similar to ACGME resident milestones [23-25].

## Conclusions

Female faculty, at the clinical instructor and assistant professor level, were rated as allowing less autonomy and providing more supervision in direct patient care and bedside procedures than their male faculty counterparts of the same rank. Evaluations of faculty teaching by learners can be used by chairs and those in leadership positions to inform promotions criteria and incentive structures only when appropriately designed. This includes consideration and mitigation of implicit biases within evaluation tools and training on evaluation scoring. It is important for any institution that utilizes teaching scores for the purposes of annual review or promotion and tenure to consider whether comparisons of an individual faculty member to the mean are statistically valid and meaningful.
